# Optimizing Identification and Care of Pregnant/Breastfeeding Women Living With HIV in Resource‐Limited Settings

**DOI:** 10.1002/jia2.70126

**Published:** 2026-06-19

**Authors:** Mary Glenn Fowler, Victoria B. Chou, Philippa Musoke

**Affiliations:** ^1^ Department of Pathology Johns Hopkins School of Medicine Baltimore Maryland USA; ^2^ Department of International Health Johns Hopkins Bloomberg School of Public Health Baltimore Maryland USA; ^3^ MU‐JHU Research Collaboration Kampala Uganda

## Abstract

**Introduction:**

Since the mid‐1990s, there have been major advances in diagnosing and providing antiretroviral therapy (ART) for pregnant women living with HIV (WLWH) in both resource‐rich and resource‐limited settings. Initial progress was based on the 1994 landmark perinatal trial, which showed 67% reduction in vertical transmission among infants born to non‐breastfeeding mothers in the United States when zidovudine was given during pregnancy, at labour/delivery and for 6 weeks after birth.

International perinatal trials began testing “short‐course” zidovudine regimens during late pregnancy and at labour/delivery to develop cost‐effective, feasible and deliverable interventions for low‐resource environments. More recent research has focused on the delivery of cost‐effective combination triple ART during pregnancy and breastfeeding for WLWH. The latest trial results indicate that providing lifetime maternal ART at the time of antenatal diagnosis improves overall survival and decreases morbidity, while reducing vertical transmission to <1%.

**Discussion:**

Translation of clinical trial results into successful widescale programme implementation remains a major challenge, particularly in low‐ and middle‐income countries with high HIV seroprevalence and weak maternal and child health infrastructure. Interventions recommended earlier in the global epidemic are no longer adequate, but key points of the perinatal cascade of services remain crucial to both ensure successful HIV care for WLWH and maximize reductions in vertical transmission. Notable progress made in Botswana and Uganda is valuable to highlight country‐led successes. Rapid HIV testing for pregnant women whose status is unknown, followed by immediate implementation of lifetime maternal ART, and linkage to long‐term HIV care/treatment are essential. Counselling on adherence, as well as the use of long‐acting ART regimens and holistic support for women's care, including psychosocial support, are also needed. A current major challenge has been the sudden reduction in international donor funding, which has had a significant negative impact on continuity and effective delivery of HIV care/treatment services.

**Conclusions:**

Much progress has been made in advancing HIV interventions and programmes for WLWH and pregnant and breastfeeding mothers. However, several challenges persist, which compromise delivery of effective interventions on the path to eliminate perinatal HIV transmission, and care/treatment remains suboptimal for WLWH, especially in resource‐limited settings.

## 1 Introduction

Clinical trial research on HIV prevention and treatment, including perinatal prevention and maternal/paediatric treatment, has seen major advances since the HIV/AIDS pandemic was first recognized in the 1980s. Worldwide, there has been steady progress in both treatment for women living with HIV (WLWH) and prevention of vertical transmission since the ACTG076 perinatal clinical trial in 1994. This landmark trial first demonstrated that zidovudine (ZDV), given during the second and third trimesters of pregnancy, at labour/delivery and as prophylaxis to the newborn, could reduce transmission about two‐thirds in a non‐breastfeeding group of US pregnant women [1].

International studies then built on this proof of concept and focused on delivery of “short‐course,” less expensive antiretroviral (ARV) interventions, starting with single ARVs given in late pregnancy or at labour/delivery combined with short‐course newborn prophylaxis. Trials of short‐course ZDV starting in late pregnancy among non‐breastfeeding WLWH in Asia were able to reduce vertical transmission ∼50% [2, 3] and a trial in Uganda reduced transmission by 47% with a single dose of nevirapine given to mothers at the onset of labour and to newborns soon after birth [4]. These trials then expanded to use dual combination ARVs such as ZDV/lamivudine [5, 6]. The primary focus of perinatal HIV trials in the mid‐1990s to early 2000s was to reduce the risk of vertical transmission to the infant [7] with ARV administration in late pregnancy and labour/delivery as the critical window [8]. There was increased emphasis in the 2000s on preventing transmission from breastfeeding, heightened by a trial in Kenya highlighting a greater risk of HIV acquisition from breastfeeding compared to formula feeding [9]. Some noted that interventions “should be concerned with the rights of both the child and the mother” [10].

From 2010 to 2016, leadership from the World Health Organization (WHO), UNAIDS and the international research community moved to advance HIV treatment to begin at the time of diagnosis in all persons living with HIV, including pregnant and breastfeeding women; and to harmonize these efforts with prevention efforts by using treatment as part of prevention. For perinatal HIV prevention, debates arose about Option A (ZDV monotherapy) versus Option B (providing ART during pregnancy and breastfeeding) and B+ (initiation of lifetime ART at time of diagnosis) among women who did not meet WHO or national criteria at that time for initiating HIV treatment. Several clinical trials subsequently demonstrated the importance of starting ART after HIV diagnosis regardless of immune or clinical status [11, 12]. The multi‐site PROMISE 1077BF trial showed that while both maternal ART and ZDV alone during pregnancy reduced transmission to ≤5% (part of the WHO goal for perinatal HIV elimination), the ART regimen was significantly more efficacious than ZDV alone, with peripartum transmission rates of 0.5% compared to 1.8% [13].

While clinical trials demonstrated that vertical transmission can be reduced and pregnant and postpartum WLWH can achieve non‐detectable viral loads and lead healthy, asymptomatic lives, efficient translation of research findings to clinical practice has been challenging. Progress has been particularly uphill in resource‐limited international settings. UNAIDS reported in 2024 [14]:

- 40.8 million (37.0–45.6 million) people globally were living with HIV.
- 1.3 million (1.0–1.7 million) people acquired HIV.
- 31.6 million (27–31.9 million) people were accessing antiretroviral therapy (ART).
- 45% of all new HIV acquisitions were among women and girls (all ages).
- In sub‐Saharan Africa (SSA), cases among women and girls (all ages) accounted for 63% of all new HIV acquisitions.

For vertical HIV transmission, UNAIDS reported that an estimated 1.1 million (0.9−1.3 million) pregnant women need ARV to prevent vertical transmission, and about a quarter of these women are young mothers (aged 15–24 years old).

- 84% of pregnant WLWH received ARV to prevent vertical transmission.
- Of the 120,000 (82,000–170,000) new infant HIV acquisitions in 2024, most (83%) were among children living in SSA.
- Since 2010, the number of new HIV acquisitions among children has declined by 62%, but 36% of all child HIV acquisition occurs in western and central Africa, where only 56% of pregnant WLWH receive ART to prevent vertical transmission.

## 2 Discussion

### 2.1 Where Are We Now?

Within this context, trends for routine HIV testing during pregnancy, uptake of ART by pregnant WLWH and vertical transmission indicate steady global gains, but rates vary substantially by region, country and factors such as rural versus urban geography. For example, as short‐course ZDV and then ART interventions became available, middle‐income countries such as Thailand [15] and Botswana [16] were early leaders implementing highly effective programmes to identify pregnant WLWH, provide high coverage for antenatal HIV diagnostic and treatment services, and ensure follow‐up to establish robust linkages for long‐term HIV care and treatment. Successful national and local intervention led to early identification of pregnant WLWH by routine testing in step with the WHO's Path to Elimination for eliminating vertical transmission of HIV [16, 17]. WHO validation requires a case rate of <250 per 100,000 livebirths for vertical transmission and ≥95% coverage for antenatal care, HIV testing and ART [18]. As of the end of 2025, 19 countries have reached successful elimination of vertical HIV transmission [19].

In countries with a high prevalence of HIV in the general population and among women of childbearing age, collaboration and commitment are essential to reach global targets. In countries with prevalent breastfeeding, such as Uganda and Kenya, HIV testing among pregnant women is high, and they are close to achieving the WHO goal of providing maternal lifetime ART at the time of diagnosis, antenatally and during breastfeeding. Several countries in eastern and southern Africa have dropped HIV vertical transmission to <5% (e.g. Botswana, Lesotho, Namibia, Eswatini, South Africa), and Botswana was the first African country with a high HIV burden to meet the WHO criteria for elimination. Western and central African countries have had less success diagnosing HIV during pregnancy, and elimination efforts are hampered by limited rapid HIV testing during pregnancy, resulting in low rates of maternal ART uptake and higher rates of vertical HIV transmission (see Table 1).

**TABLE 1:** HIV/AIDS indicators among representative sub‐Saharan African countries.

Country	HIV prevalence among pregnant women^a^	Percentage of pregnant women living with HIV accessing antiretroviral medicines (2024)^b^	Vertical transmission rate (2024)^b^	Early infant diagnosis (2023)^b^
Botswana	24%	>98% [91%−>98%]	1% [1%−3%]	86% [77%−112%]
Burkina Faso	1%	69% [58%−81%]	16% [13%−20%]	19% [17%−23%]
Cameroon	6%	59% [52%−72%]	15% [13%−17%]	65% [57%−79%]
Cote d'Ivoire	9%	88% [73%−>98%]	9% [4%−15%]	61% [54%−74%]
Kenya	7%	90% [77%−>98%]	9% [7%−13%]	82% [71%−97%]
Malawi	10%	96% [88%−>98%]	7% [6%−8%]	85% [78%−100%]
Nigeria	7%	33% [30%−40%]^c^	23% [21%−26%]^c^	18% [16%−22%]
South Africa	29%	>98% [81%−>98%]	3% [2%−5%]	90% [75%−125%]
Uganda	7%	>98% [97%−>98%]	6% [5%−8%]	82% [74%−95%]
Zimbabwe	16%	93% [84%−>98%]	6% [4%−8%]	84% [75%−100%]

^a^ See References [20, 21, 22, 23, 24, 25, 26, 27, 28, 29].

^b^ 2024 Estimates from: UNAIDS Global AIDS Update 2025. Geneva: Joint United Nations Programme on HIV/AIDS; 2025. License: CC BY‐NC‐SA 3.0 IGO via https://aidsinfo.unaids.org/.

^c^ 2023 Estimates from: UNAIDS Global AIDS Update 2024. Geneva: Joint United Nations Programme on HIV/AIDS; 2024. License: CC BY‐NC‐SA 3.0 IGO.

Differences across region and country in resource‐limited settings with high HIV prevalence are related to multiple factors that impact the “perinatal cascade” of HIV services, which is necessary to ensure optimal care for WLWH as well as maximal reduction of new infant HIV acquisitions. The Perinatal Cascade of HIV Prevention and Treatment Delivery of Services ideally includes:

- Preconception and/or prenatal HIV counselling with emphasis on Pre‐exposure Prophylaxis (PrEP) strategies for women of unknown or HIV‐negative status; and adherence for WLWH, along with disclosure and couple counselling.
- Test and treat during pregnancy and post‐delivery based on country and WHO guidelines.
- Adherence counselling for ART for WLWH; and follow‐up appointments antenatally and postpartum for both WLWH and HIV‐negative mothers.
- Counselling on the importance of exclusive breastfeeding during the first 4–6 months postpartum.
- Repeat HIV testing during breastfeeding for mothers with negative or unknown HIV status, according to country and WHO guidelines.
- Newborn and infant HIV nucleic acid testing, with final testing following cessation of breastfeeding; and careful monitoring of growth and development.

Current emphasis is on PrEP interventions to reduce the risk of new maternal acquisition of HIV during pregnancy and during breastfeeding, which globally are contributing to the majority of new cases of infant HIV acquisition.

Key elements of the perinatal cascade of HIV services must be in place to ensure comprehensive HIV care and treatment services starting from the determination of HIV status for pregnant and breastfeeding women, and providing effective early HIV treatment for those tested and found to be WLWH, along with PrEP services for at risk HIV‐negative women as needed. This requires integration across the healthcare system and optimized quality of care, with routine rapid HIV testing for all pregnant women at their first prenatal booking, followed by repeat testing in the third trimester, at 6 weeks post‐delivery, and every 3 months during breastfeeding. For newly diagnosed pregnant or breastfeeding WLWH, immediate initiation of lifetime HIV ART aligned with the country's current Standard of Care policies is critical. During breastfeeding, continuity must be sustained to ensure that recommended maternal HIV testing is repeated for incident HIV acquisitions. In addition to counselling to promote ART adherence and HIV care follow‐up, maternal education about breast care to prevent mastitis and/or breast abscess, and exclusive breastfeeding promotion for the first 4–6 months of life are also important to reduce the risk of HIV transmission during breastfeeding.

Unfortunately, numerous obstacles can interfere with optimal care for pregnant and breastfeeding WLWH in international settings. These include societal and cultural issues, structural factors, as well as national commitment to provide adequate funding for maternal/child health services. Global disruptions due to armed conflicts, civil unrest and funding interruptions profoundly impact health and the sudden withdrawal of international HIV funding starting in 2025 adversely affected many aspects of the perinatal HIV prevention cascade [19], and sub‐Saharan African countries may be disproportionately affected [30].

At an individual level, WLWH who become pregnant may be especially vulnerable due to young age or lack of agency, which interferes with a woman's ability to adhere to HIV care and treatment guidelines. HIV stigma may also play a role and result in a lack of disclosure to family members, which can affect medication adherence and follow‐up care. Pregnancy and postpartum have been identified as particularly difficult periods for maternal adherence to ARV drug regimens [31].

At regional and national levels, challenges include inadequately resourced health facilities and periodic stockouts of essential clinic supplies, including rapid testing kits and HIV drugs. National guidelines for repeat testing of breastfeeding women are often not widely followed, resulting in fragmented or substandard quality of care. Government funding for maternal/child health (MCH) services, including repeat HIV testing during breastfeeding, is typically sparse, and improved integration is key to strengthen HIV care/treatment for newly diagnosed WLWH [32]. Creating linkages can reduce missed opportunities and identify the 2%−3% of mothers without HIV in high‐prevalence settings who seroconvert after delivery [33].

### 2.2 Lessons Learned From Botswana, an African Country That Has Eliminated Vertical HIV Transmission

As a landlocked country in southern Africa, Botswana is a middle‐income country with a high prevalence of HIV that has overcome poverty and inequality barriers to strengthen data and laboratory capacity and improve primary healthcare. Annual case rates for new HIV paediatric acquisitions declined from <500 to <250 per 100,000 livebirths when national stakeholders, including the Ministry of Health (MoH), committed to increasing coverage to 95% for antenatal care, HIV testing and treatment of pregnant WLWH. They met the stringent WHO criteria for eliminating vertical transmission of HIV despite the country's high burden of HIV [16]. Factors leading to their remarkable success included political will and in‐country funding, early adoption of new research findings and rapid translation into policy, and key investments to improve integrated care. Botswana now continues towards “triple elimination” by targeting the elimination of syphilis and hepatitis B virus transmission as the next public health priorities.

### 2.3 Lessons Learned From Uganda, an African Country Close to Eliminating Vertical HIV Transmission

As a sub‐Saharan African country heavily affected by the HIV pandemic [34], Uganda has been a leader in perinatal research, and since introducing single‐dose nevirapine to prevent vertical transmission [4], there has been significant progress in the scale‐up of perinatal HIV services. Prevalence of maternal HIV has decreased from ∼30% in the 1990s to 7% in 2024 among pregnant Ugandan women. Vertical transmission has likewise decreased from 25% to 30% in 2000% to 6% in 2024 [14].

High rates of vertical transmission in Uganda in the 1990s were primarily due to the lack of ART for pregnant WLWH and prolonged infant breastfeeding. Uganda adopted WHO guidelines [35] and implemented triple therapy to prevent vertical transmission in 2016, with the implementation of dolutegravir‐based ART for pregnant and breastfeeding women in 2018 [36]. Uganda's success can be attributed to sustained MoH commitment since the early 2000s, technical guidance from Ugandan researchers, as well as international donor funding. However, the country has not yet achieved the elimination of vertical transmission (<5%) in a breastfeeding population [14].

The perinatal cascade of HIV services ensures that all (>99%) pregnant women attending antenatal care are screened for HIV with same‐day results, enabling initiation of triple therapy for those living with HIV. Currently, >95% who test positive start triple ART and for pregnant women who complete this cascade, vertical transmission is <2%. Most new paediatric HIV acquisitions occur in women who seroconvert (26%) or discontinue ART (52%) during pregnancy; with many seroconverting (43%) and discontinuing ART (42%) during breastfeeding [37], with treatment fatigue, non‐disclosure, stigma, distance from health unit and ART side effects cited as contributing factors [38].

Follow‐up during pregnancy and breastfeeding is crucial, and Uganda's guidelines recommend repeat HIV testing every 3 months for pregnant and breastfeeding women who test HIV negative [36], but adherence to repeat HIV testing across the continuum of care is limited. New strategies with long‐acting injectables, including cabotegravir and lenacapavir, for pre‐exposure prophylaxis may critically reduce the risk of HIV acquisition in these vulnerable populations [39, 40, 41].

Both Botswana and Uganda reflect strong success in both perinatal HIV prevention and maternal HIV care and treatment. Botswana has relied heavily on in‐country financing and early internal adoption of national policies to ensure adequate funding for ART treatment and vertical HIV elimination programmes without dependence on external donor support. Uganda, a more resource‐limited country, has depended more on external donor funding but has been bolstered by strong commitment from the MoH and technical input from in‐country researchers. Both countries have had strong advocacy from national HIV researchers and expanded to reach both rural and urban areas with frontline healthcare workers and community‐based programmes, as well as strengthened supply chain systems, to deliver integrated HIV perinatal and maternal treatment and HIV prevention services through the existing MCH and public health platforms.

### 2.4 Ways Forward

Lessons learned from Botswana and Uganda underscore the importance of strong local and national commitment to support HIV care programmes for pregnant and breastfeeding mothers. Success requires MoH leadership, partnership with international funders, and substantial technical input and advocacy from researchers leading and conducting HIV‐related treatment and prevention trials. Further optimizing to support pregnant and breastfeeding women will require:

- Sustained *commitment from governments* in resource‐limited settings to prioritize strengthening MCH infrastructure to deliver the perinatal cascade of HIV services, which ensures pregnant WLWH are identified and linked to lifetime ART care.
- Identifying alternative *funding* given the uncertainty/fragility of international aid and donor funding. Costs for maintaining vital ART programmes must be balanced amidst new funding needs for primary prevention scale‐up, including long‐acting ARVs for pre‐exposure prophylaxis.
- Addressing identified weaknesses in the *perinatal cascade* of HIV services, including immediately post‐delivery and during breastfeeding. Examples include use of long‐acting ARV interventions to reduce the risk of new maternal sero‐incident acquisitions in late pregnancy and postpartum; early identification of maternal seroconversions postpartum; as well as strengthening interventions to support maternal ART adherence, particularly postpartum when levels may drop, and loss to MCH follow‐up frequently occurs.
- Continued sponsorship for *ongoing research* to study long‐acting ARV drugs and develop novel immune and other innovative approaches for HIV treatment/prevention.
- Partnerships to ensure *access/availability* of new ART drugs at affordable prices for both treatment for WLWH and primary prevention among HIV‐negative adolescents and women at risk.
- *Political will and continued advocacy* by women's groups, government agencies and international health agencies to prioritize the health and wellbeing of WLWH. HIV care and treatment interventions are the first building block for cost‐effective triple elimination efforts as we move beyond HIV towards prevention and treatment of maternal hepatitis, syphilis and congenital infections.

## 3 Conclusions

The world is at a difficult crossroads in terms of the HIV/AIDS pandemic. Major progress since the 1990s has resulted in highly effective regimens for both treatment and primary prevention—with long‐acting drugs showing great promise to further optimize treatment for pregnant and breastfeeding WLWH. However, translation of research and progress to scale‐up in resource‐limited settings may be stalled due to the withdrawal of international funding and reduced support for non‐governmental organizations supporting crucial implementation efforts. Strong advocacy at the local, national, and international levels and innovative approaches to advance cost‐effective HIV care and treatment for pregnant and breastfeeding WLWH are needed to overcome current challenges.

## Author Contributions

The first draft of this commentary was developed by MGF and PM. MGF led manuscript development with contributions and input from PM and VBC. All authors contributed to the final manuscript and approved it for publication.

## Disclaimer

The conclusions and opinions expressed in this article are those of the authors and do not necessarily reflect those of the National Institutes of Health or the U.S. Department of Health and Human Services.

## Conflicts of Interest

The authors have no conflicts of interest to disclose.
